# An epidemiological study of *Streptococcus suis* prevalence among swine at industrial swine farms in Northern Vietnam

**DOI:** 10.1016/j.onehlt.2021.100254

**Published:** 2021-04-24

**Authors:** Nguyen Thao Thi Nguyen, Yen Thi Hai Luu, Trung Duc Hoang, Huyen Xuan Nguyen, Tung Duy Dao, Vuong Nghia Bui, Gregory C. Gray

**Affiliations:** a Duke University School of Medicine, Duke University, Durham, NC 27710, United States; b Bacteriology Department, National Institute of Veterinary Research, Hanoi 100000, Viet Nam; c Virology Department, National Institute of Veterinary Research, Hanoi 100000, Viet Nam; d Duke Global Health Institute, Duke University, Durham, NC 27710, United States; e Emerging Infectious Diseases Programme, Duke-NUS Medical School, Singapore; f Global Health Center, Duke Kunshan University, Kunshan, China

**Keywords:** *Streptococcus suis*, Swine, Swine farms, Swine workers, Bioaerosol

## Abstract

**Introduction:**

*Streptococcus suis* is a zoonotic pathogen found in swine that may cause systemic infection in humans. *S. suis* is endemic in Southeast Asia and is the leading cause of adult meningitis in Vietnam. Given Vietnam's increasing centralization of the swine industry, we sought to estimate the prevalence of *S. suis* on large swine farms in Northern Vietnam.

**Methods:**

A cross-sectional, one-health-oriented, surveillance study for *S. suis* was conducted between October 2019–March 2020. Swine oral, swine worker nasal, and bioaerosol samples were collected from four large-scale swine farms (>500 swine) in three provinces in Northern Vietnam: Lao Cai, Bac Giang, and Quang Ninh. Samples were evaluated for presence of *S. suis* growth on blood agar plates and confirmed with conventional polymerase chain reaction.

**Results:**

The authors found that 4/174 (2.3%, 95% CI: 0.6–5.8%) of swine oral samples and 1/58 (1.7%, 95% CI: 0–9.2%) bioaerosol samples were positive for *S. suis* by bacterial culture and conventional PCR. *S. suis* was not detected in any swine worker nasal wash samples. There was no significant relationship between sampling location and month of sample collection with results of swine oral or bioaerosol samples.

**Conclusion:**

Compared to previous reports from slaughterhouses in Vietnam, the lower than expected prevalence of *S. suis,* supports the notion that that recent efforts to centralize Vietnam's pork industry through establishment of large-scale farms with better biosecurity may have been effective in limiting *S. suis* prevalence on these large farms.

## 1 Introduction

*Streptococcus suis* is a zoonotic pathogen found in swine that may cause systemic infection in humans—most commonly meningitis. One distinct feature of *S. suis* meningitis is the hearing loss that is reported in up to one half of infected patients [1,2]. Other clinical manifestations of *S. suis* infection include endocarditis, pyogenic arthritis, endophthalmitis and uveitis, spondylodiscitis, brainstem ophthalmoplegia, and epidural abscess [3].

*S. suis* resides in the upper respiratory tract of swine, in addition to the genitourinary and gastrointestinal tracts [4]. Thus far, transmission to humans is believed to be primarily through cutaneous infection through open wounds and oral ingestion of undercooked meat [5]. However, a recent study conducted in swine confinement buildings in Canada has also suggested that *S. suis* can be transmitted through aerosolization [6].

While human infection by *S. suis* was first reported in 1968 in Denmark, the disease has since been found to be more prevalent among East and Southeast Asian countries, accounting for more than 90% of all reported cases globally [7]. The high rates of *S. suis* meningitis in Vietnam are likely related to the country's substantial swine industry, which was documented to produce more than 26 million swine in 2013, along with regional practices that result in higher levels of human exposure to swine [8].

Historically, household producers accounted for 90% of the Vietnam's pork supply with swine production contributing to 9–41% of their total income [9,10]. However, Vietnam's pork industry has evolved in the past decade towards a more centralized system of pork production—changes driven by both economic profitability and increasing concerns related to disease transmission [11]. Indeed, the national government has encouraged this transition on the assumption that industrialized production would support improved disease control [12,13].

Due to the country's increasing centralization of the swine industry, we focused on large-scale swine farms to further evaluate the effectiveness of disease control among industrialized farms. Accordingly, this study sought to determine the prevalence of *S. suis* among swine, swine workers, and bioaerosol environments among large-scale swine farms in Northern Vietnam with the aim of shedding light on prevalence of *S. suis* colonization and possible transmission risk to humans.

## 2 Methods

A cross-sectional surveillance study for *S. suis* at four large-scale swine farms in three provinces was conducted between October 2019–March 2020. This study was conducted in conjunction with Duke One Health and the National Institute of Veterinary Research (NIVR) in Vietnam as a part of their larger project, “Pathogen Surveillance among Pigs in Vietnam.” Permission to sample from swine farms was granted through NIVR's national-level approval from the Ministry of Agriculture and Rural Development's Department of Animal Health. Swine farm operators also gave verbal permission to allow sampling at their sites. The swine sampling protocol was approved by the Duke University Institutional Animal Care and Use Committee. No invasive studies of animals were performed. Human survey and sampling protocols were approved by the Institutional Review Board at Duke University and Ministry of Health Vietnam.

### 2.1 Sample collection

**Fig. 1:**
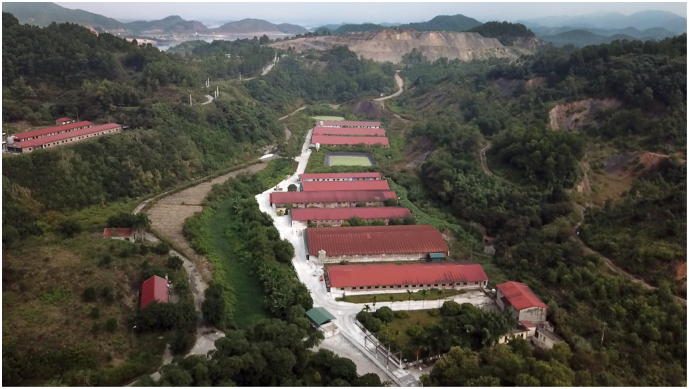
Aerial view of swine farm in Quang Ninh province Photo by Jim Rogalski.

Samples were collected from four large-scale swine farms (>500 swine) in three provinces in Northern Vietnam: Lao Cai, Bac Giang, and Quang Ninh (Fig. 1). Samples were collected approximately once per month in each province.

Three types of samples were collected at swine farms—swine oral, swine worker nasal, and bioaerosol. Because this study was conducted shortly following the African Swine Fever epidemic in 2019, farms did not allow visitors to enter the swine farms. Farm staff were trained to conduct swine oral and bioaerosol sampling with video monitoring by researchers to ensure consistency of sampling techniques. Informed consent and nasal sampling were collected outside of swine farm premises by research staff.

Swine oral samples were collected using a non-invasive rope-sampling in which sterile ropes were hung in strategic areas around swine farms on which swine could chew. After the 30-min sampling period, ropes were saturated with swine oral fluids. These fluids were extracted by squeezing the rope to expel the collected oral fluids into a sterile container. A prior study in Korea has demonstrated the efficacy of using rope-sampling with polymerase chain reaction (PCR) analysis for surveillance of porcine respiratory diseases and detected the presence of *S. suis* in 56% of pens. [14] Another study in Vietnam found the abundance of *S. suis* in pig saliva, suggesting that oral salivary samples are sufficient to detect the pathogen. [15] Indeed, in settings where access to veterinary services is limited, rope sampling in pens has demonstrated comparable detection of respiratory pathogens as compared to sampling of individual swine. [16] Ultimately, non-invasive rope-sampling was chosen in this study for its non-invasiveness and reproducibility.

**Fig. 2:**
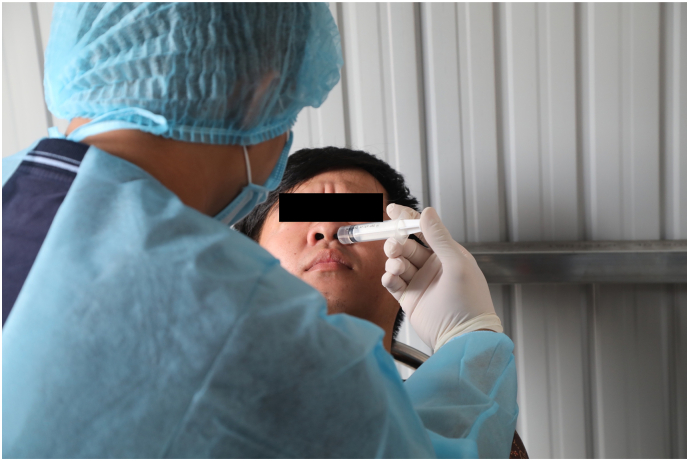
Nasal wash collection at Quang Ninh swine farm Photo by Jim Rogalski with verbal consent by participant.

At each participating farm, swine workers were invited to participate in the study when the study team made their visit (convenience sample), offered a modest nonmonetary incentive, and most participated. After written informed consent was obtained, nasal wash samples were collected. Participants were asked to tilt their head back and briefly hold their breath while one nostril was irrigated with 5 mL of sterile water (Fig. 2). The participant then expressed the fluid into a sterile collection cup. This was repeated for the other nostril. Demographics of participants including sex, age, and ethnicity were collected. Some participants were asked to participate more than once during the 5-month study period.

Bioaerosol samples were collected using four National Institute for Occupational Safety and Health (NIOSH) 2-stage aerosol samplers. These samplers ran for 3 h at a flow rate of 5 L/min during each farm visit [17]. Samplers were evenly distributed throughout swine areas and were fixed above the ground on a stationary tripod. Bioaerosol samples were captured in a 15-mL conical tube, 1.5-mL centrifuge tube, and a polytetrafluoroethylene filter cassette attached to each NIOSH sampler. After collection, all samples were appropriately labeled, preserved in portable liquid nitrogen tanks, and transported back to NIVR.

At NIVR, the 15-mL conical tubes and 1.5-mL centrifuge tubes were detached from the NIOSH samplers, after which 2 mL of sterile collection medium (phosphate-buffered saline with 0.5% wt/vol bovine serum albumin fraction V) was added to each 15-mL centrifuge tube and 1 mL of collection medium was added to each 1.5-mL centrifuge tube. Sampler tubes were then vortexed and then transferred to 2.0-mL cryovial tubes. Filter cassettes were then removed from the NIOSH samplers and each polytetrafluoroethylene filter was transferred to the bottom of a 50-mL conical tube and vortexed for 15 s while dry. Next, 1 mL of collection medium was added to each 50-mL tube and vortexed twice before removing and discarding the filter. The vortexed sample solutions in the 50-mL tubes were then transferred to the 2.0-mL cryovial tubes containing the sample solutions from the 1.5-mL centrifuge tubes previously detached from the NIOSH samplers, yielding a 2-mL combined sample tube.

Tubes containing swine oral and human nasal samples were vortexed twice at medium speed. The remaining solution was transferred to cryovial tubes and divided into 2 mL aliquots. Bioaerosol, swine oral, and human nasal samples were stored at 4 °C for further processing.

### 2.2 Isolation of *S. suis*

Swine oral, human nasal, and bioaerosol samples were centrifuged at 3000 rpm for 2 min. Samples were directly plated onto blood agar plates containing 5% sheep blood and incubated at 37 °C for 24 h. At the same time, 100 μl of the specimen was also incubated in 4 ml of tryptic soy broth (TSB) supplemented with 5% Fildes enrichment. If there was insufficient growth with direct plating, TSB-incubated specimen was used to re-plate on blood agar plates and re-evaluate for growth. After incubation, plates were evaluated for *Streptococcus*-like growth. Colonies resembling *Streptococcus* (small, gray or transparent, mucoid, α or α to β hemolytic colonies) were transferred to another blood agar plate supplemented with 5% sheep blood to further incubate overnight [18]. Isolated colonies were then confirmed with Gram staining. Colonies identified to be Gram-positive cocci were regrown on blood agar plates again in preparation for identification with conventional PCR [19]. PCR analysis was only performed on colonies that grossly and microscopically resembled *S. suis* to minimize contamination with *S. suis* molecular material derived from nonviable bacteria. [20] Real-time PCR was not used due to resource limitations in the local laboratory setting.

### 2.3 Molecular identification of *S. suis*

One μL of isolated bacterial colonies were suspended in 100 μL of nuclease-free water and heated at 100 °C for 20 min in a heating block to disrupt cell walls and denature remaining membranes, releasing nucleic acid for amplification. Afterwards, the tubes were immediately placed on ice for 5 min. Colonies were then centrifuged at 13,000 rpm for 2 min. The supernatant containing nucleic acid was then extracted for conventional PCR analysis.

Molecular identification of *S.* suis was determined using conventional PCR with the glutamate dehydrogenase (*gdh*) gene, resulting in a 688 base pair fragment. Prior studies have demonstrated that amplification of the *gdh* gene can effectively identify all *S. suis* serotypes types across diverse organisms and geographic regions with 100% sensitivity and specificity. [21] The PCR primers used were JP4 (5′-GCAGCGTATTCTGTCAAACG-3′) and JP5 (5′-CCATGGACAGATAAAGATGG-3′) (Oxford Molecular Group, Inc., Campbell, CA, USA). The PCR thermal cycle profile comprised of 5 min at 94 °C, followed by 35 cycles of 1 min at 94 °C, 1 min at 55 °C, 1 min at 72 °C, and 7 min at 72 °C. [21] PCR products were evaluated using gel electrophoresis and visualized with UV light. Size of DNA products was determined by comparison with a DNA ladder. Samples that demonstrated DNA products approximately 688 base pairs in size were considered positive for *S. suis* in this study. Amplified products were not sequenced in this study due to resource limitations though future studies should consider sequencing to further characterize the isolated bacteria.

### 2.4 Statistical analysis

The data were imported into RStudio version 1.2.5033 (RStudio, Boston, MA, USA), cleaned, and categorized for statistical analyses. For participant demographic data, continuous data were categorized (*e.g.*, age in decades) for ease of study. Descriptive statistics was used to characterize data frequency, percent, mean, and standard deviation. Confidence intervals were calculated based on a on a confidence level of 95%. For subgroups in which the incidence was zero, the rule of three was used in which maximum risk was estimated to be *3/n* [22]. Fisher's exact tests were used to examine independence between categorical variables. Statistical significance was defined at a *p*-value of less than 0.05.

## 3 Results

As demonstrated by Table 1, samples were collected from large-scale swine farms across three provinces in Northern Vietnam. A total of 174 swine oral, 116 swine worker nasal wash, and 58 bioaerosol samples were collected. Of these, four (2.3%, 95% CI: 0.6–5.8%) swine oral samples and one (1.7%, 95% CI: 0–9.2%) bioaerosol sample were positive for *S. suis* by culture and then confirmed by PCR. Of the 4 positive swine oral samples, three were collected from Quang Ninh province, and one was from Bac Giang province. The positive bioaerosol sample was from Bac Giang province. *S. suis* was not detected in any of the swine worker nasal wash samples (95% CI: 0–3%) [22]. There was no significant difference in prevalence of *S. suis* in swine oral and bioaerosol samples.

**Table 1:** Prevalence of *S. suis* in swine oral, human nasal, and bioaerosol samples.

Sampling location	Collection date	Proportion positive		
		Swine oral	Swine worker nasal	Bioaerosol
Bao Thang, Lao Cai	25 Oct 2019	0/12	0/8	0/4
	23 Nov 2019	0/12	0/8	0/4
	28 Dec 2019	0/12	0/8	0/4
	17 Jan 2020	0/12	0/8	0/4
	28 Feb 2020	0/12	0/8	0/4
	24 March 2020	0/12	0/8	0/4
	**Total**	**0/72 (0%)**	**0/48 (0%)**	**0/24 (0%)**
Hiep Hoa, Bac Giang	31 Oct 2019	0/12	0/8	0/4
	27 Nov 2019	0/12	0/8	1/4
	25 Dec 2019	0/12	0/8	0/4
	14 Jan 2020	0/12	0/8	0/4
	21 Feb 2020	0/12	0/8	0/4
	11 Mar 2020	1/12	0/8	0/4
	**Total**	**1/72 (1.4%)**	**0/48 (0%)**	**1/24 (4.2%)**
Cam Pha, Quang Ninh	15 Nov 2019	2/6	0/4	0/2
	6 Dec 2019	0/6	0/4	0/2
	6 Jan 2020	0/6	0/4	0/2
	9 Feb 2020	1/6	0/4	0/2
	9 Mar 2020	0/6	0/4	0/2
	**Total**	**3/30 (10%)**	**0/20 (0%)**	**0/10 (0%)**
All provinces	Proportion positive (%)	4/174 (2.3%)	0/116 (0%)	1/58 (1.7%)
	95% CI	0.6–5.8%	0–3%	0–9.2%

Table 2 demonstrates test for independence between sampling location and month in which sample was collected and results of swine oral and bioaerosol samples. As shown, there was no significant association found between sampling location and month of sample collection and swine oral or bioaerosol results.

**Table 2:** Tests for independence of variables.

Swine oral		Proportion positive	*p*-value
Sampling location	Bao Thang, Lao Cai	0/72	0.12
	Hiep Hoa, Bac Giang	1/72	
	Cam Pha, Quang Ninh	3/30	
Month	October	0/24	0.71
	November	2/30	
	December	0/30	
	January	0/30	
	February	1/30	
	March	1/30	

Bioaerosol		Proportion positive	*p*-value
Sampling location	Bao Thang, Lao Cai	0/24	1
	Hiep Hoa, Bac Giang	1/24	
	Cam Pha, Quang Ninh	0/10	
Month	October	0/8	1
	November	1/10	
	December	0/10	
	January	0/10	
	February	0/10	
	March	0/10	

Table 3 demonstrates the demographics of swine worker participants in this study. This study collected 116 nasal wash samples from 74 unique individuals. The majority of participants were male (62.2%), were between 20 and 29 years of age (47.3%), and identified with the Kinh ethnic group (64.9%). Thirty participants participated in this study more than once, with 18 participating twice and 12 participating three times. Among those who participated more than once in this study, 12 (40.0%) were from Lao Cai, 11 (36.7%) were from Bac Giang, and 7 (23.3%) were from Quang Ninh provinces.

**Table 3:** Swine worker demographics.

Characteristics		*n*	%
All participants (*n* = 74)			
Sex	Male	46	62.2%
	Female	28	37.8%
Age (years)^a^	< 20	6	8.1%
	20–29	35	47.3%
	30–39	8	10.8%
	40–49	11	14.9%
	50–59	8	10.8%
	> 60	6	8.1%
Ethnicity	Kinh	48	64.9%
	Tay	11	14.9%
	Thai	7	9.5%
	Dao	5	6.8%
	Nung	3	4.1%
Duplicate participants (*n* = 30)			
Sampling location	Bao Thang, Lao Cai	12	40.0%
	Hiep Hoa, Bac Giang	11	36.7%
	Cam Pha, Quang Ninh	7	23.3%

a Age at time of study enrollment.

## 4 Discussion

This study estimated the prevalence of *S. suis* among swine, swine workers, and swine farm bioaerosol environment among four large-scale swine farms in Northern Vietnam. In summary, 4/174 (2.3%, 95% CI: 0.6–5.8%) of swine oral samples and 1/58 (1.7%, 95% CI: 0–9.2%) bioaerosol samples were found to be positive for *S. suis* by conventional PCR. *S. suis* was not detected in any swine worker nasal wash samples. There was no significant relationship between sampling location and month of sample collection with results of swine oral or bioaerosol samples.

Our findings highlight the overall low prevalence of *S. suis* prevalence among swine, swine farm bioaerosol environments, and swine workers. In comparison to other studies in Southeast Asia where the prevalence of S. suis in swine was documented to be as high as 41% [[23], [24], [25]], the prevalence of swine colonization with *S. suis* in this study is remarkably low. This may be due to variations in sampling techniques, with previous studies using specimen collected from swine tonsils and salivary glands. Nonetheless, the efficacy of non-invasive sampling using oral fluid secretions to characterize *S. suis* colonization of swine has been previously demonstrated [14], and as such, and we believe these prevalence counts truly reflect lower rates of swine colonization at farms sampled in this study compared to previous reports. However, one limitation to our rope sampling technique is decreased precision in determining the number of swine colonized as multiple swine would chew on the same rope and some swine did not chew on any ropes at all.

The prevalence of *S. suis* among bioaerosol samples in this study was also lower than the previously reported prevalence among swine confinement buildings in Canada [6], likely reflecting the lower prevalence of overall swine colonization. Though *S. suis* prevalence was higher in swine oral and bioaerosol samples, we were unable to detect a significant difference between the two sample types. However, previous *in vitro* studies have found an overall resistance of *S. suis* to aerosolization in comparison to other common colonizers of the swine oropharyngeal tract, potentially due to its thick capsule rich in sialic acid [26,27]. Further, *in vitro* studies have also demonstrated that when *S. suis* does become aerosolized, it is more likely to retain its bacterial integrity—that is, its ability to grow and reproduce [26]. Indeed, the protocol implemented in this study required that bacteria demonstrate growth and replicability in order for a sample to be identified as positive. Thus, while there was a low prevalence of *S. suis* in bioaerosol samples in this study, these results are significant because they represent bacteria suspended in air capable of causing human infection.

Given the low prevalence of *S. suis* aerosolization in combination with stringent personal protective equipment (PPE) policies at large-scale swine farms, particularly influenced by the recent African Swine Fever epidemic, the absence of detectable *S. suis* in swine worker nasal wash samples was to be expected. These results echo negative findings from a study of swine workers in Thailand, despite high rates of swine colonization [24]. However, they differ from the study of Canadian swine confinement buildings in which 14/21 swine workers were found to have *S. suis* colonization of their nares [6]. While these discrepancies may be mediated by a variety of factors, there has been evidence that availability and adherence to PPE, in particular, may play an important role in rates of human colonization.

Though the relationship between human colonization with *S. suis* and adherence to PPE has not been explored, previous research has found that swine workers wearing face masks were less likely to have antibiotic-resistant, livestock-associated *S. aureus* colonization of their nares. Moreover, this association extended to their family members as well, in which relatives of swine workers wearing face masks were less also less likely to be colonized [28]. Aside from differences in rates of aerosolization, *S. suis* is expected have similar infectivity once aerosolized [26]. Regardless, in addition to exploring the effects of PPE on human S. suis infection, future research should also take into account the effect of other contributing factors including concentration of aerosolized bacteria, type of ventilation system, and length of exposure.

Finally, we found no relationship between month of sample collection or sampling location and positivity of bioaerosol and swine oral samples. Previous studies have demonstrated increased *S. suis* human infections during warm and rainy seasons, potentially due to warmth and humidity providing more favorable conditions for bacterial growth [29,30]. However, because the rainy season in Hanoi typically spans from May to October, the time frame of this study, conducted from October to March, limited our ability to explore this relationship. Moreover, the aforementioned studies evaluated incidence of *S. suis* among the population, and as such, it has been hypothesized that this observed effect may be mediated by increased bacterial contamination at fresh meat markets. Further studies specifically among swine workers are needed to determine the role of heat and humidity on *S. suis* in swine farm settings.

### 4.1 Limitations

Our study had a number of limitations. It was a cross-sectional study with data collected during only a brief period of the year which may have missed seasonal variation in *S. suis* prevalence. Given that the Vietnam swine population was recently affected by the African Swine Fever epidemic, sanitation, PPE, and regular symptom monitoring among swine were tightly enforced. As such, *S. suis* prevalence statistics found in this study may be lower than previous reports from Vietnam's swine industry. Further, swine farms were selected based on their receptiveness to research participation and therefore, may represent farms with greater levels of biosecurity. Further, volunteer bias was possible in the participant recruitment process; we have attempted to avert this by ensuring confidentiality of nasal wash results.

## 5 Conclusions

Overall, the low prevalence of *S. suis* in this study supports the notion that recent efforts to centralize Vietnam's pork industry through the establishment of large-scale farms and increased regulation may have been effective in limiting *S. suis* exposure to humans in these settings. Indeed, no swine workers were found to be colonized with *S. suis* in this study despite limited positive swine oral and bioaerosol samples. These results might be explained by the greater capacity of large-scale swine farms to implement more intensive biosecurity measures to prevent disease transmission between animals and humans—measures that have been augmented since the recent African Swine Fever pandemic in 2019 [31]. Small-scale swine farms, on the other hand, have been found to have less rigorous biosecurity measures in place, with previous studies documenting unsanitary swine living conditions and low rates of PPE adherence among staff [32]. These variations in levels of regulation adherence are likely due to differences in resource availability as small-scale farms have lower amounts of discretionary income to invest in disease surveillance and prevention. Moving forward, given that household producers make up a significant portion of Vietnam's pork supply chain, future research will be needed to estimate *S. suis* prevalence among smaller farms and identify barriers to appropriate sanitation and PPE adherence among these settings.

## CRediT authorship contribution statement

**Nguyen Thao Thi Nguyen:** Conceptualization, Methodology, Investigation, Formal analysis, Writing - original draft. **Yen Thi Hai Luu:** Methodology, Investigation, Writing - review & editing, Supervision, Project administration. **Trung Duc Hoang:** Investigation. **Huyen Xuan Nguyen:** Methodology, Resources, Supervision. **Tung Duy Dao:** Methodology, Investigation, Resources, Supervision, Project administration. **Vuong Nghia Bui:** Supervision, Project administration, Resources. **Gregory C. Gray:** Methodology, Resources, Writing - review & editing, Supervision, Funding acquisition.

## Declaration of Competing Interest

The authors have no conflicts of interest to declare.
